# Enhancing Cancer Truth-Telling Perspectives Using Virtual Reality in Communication Skills Training: An Experimental Study Among Medical Students

**DOI:** 10.5334/pme.1684

**Published:** 2025-09-10

**Authors:** Shih-Ying Chen, Ji-Tseng Fang, Ming-Ju Hsieh, Che-Wei Lin, Heng-Hsin Tung, Maiko Fujimori, Woung-Ru Tang

**Affiliations:** 1 School of Nursing, Chang Gung University and Division of Hematology-Oncology, Department of Internal Medicine, Linkou Chang Gung Memorial Hospital, Taoyuan, Taiwan; 2 Kidney Research Center, Department of Nephrology, Linkou Chang Gung Memorial Hospital and College of Medicine, Chang Gung University, Taoyuan, Taiwan; 3 Division of Thoracic Surgery, Linkou Chang Gung Memorial Hospital and College of Medicine, Chang Gung University, Taoyuan, Taiwan; 4 Department of Emergency, Taipei Medical University Shuang Ho Hospital, Taipei, Taiwan; 5 Department of Medical Education and Humanities, School of Medicine, College of Medicine, Taipei Medical University, Taipei, Taiwan; 6 Department of Nursing, National Yang-Ming Chiao Tung University, Taipei, Taiwan; 7 Section of Psychological Science, Division of Health Care Research/Section of Behavior Research, Division of Behavioral Science, Behavioral Sciences and Survivorship Research Group, Center for Public Health Sciences, National Cancer Center, Tokyo, Japan; 8 School of Nursing, Chang Gung University and Kidney Research Center, Department of Nephrology, Linkou Chang Gung Memorial Hospital, Taoyuan, Taiwan

## Abstract

**Introduction:**

Virtual Reality (VR) has advanced in medical education, offering enhanced realism and immersion, allowing students to safely practice complex and rare scenarios like cancer truth-telling. This study aimed to develop and evaluate the effectiveness of a VR-based cancer truth-telling module.

**Methods:**

This experimental study randomly assigned fifth-year medical students to the following groups: in-person communication skills training (iCST), VR, and a combined iCST+VR group. The intervention included a 30-minute video-based mini-lecture followed by practical application. The VR group received the VR truth-telling module, the iCST group received the small-class iCST module, and the iCST +VR group received both the iCST and VR modules. Primary outcomes assessed were cancer truth-telling perspectives, with communication confidence and learning satisfaction as a secondary outcome. Data were collected at baseline, immediately after, and at three and six months post-intervention.

**Results:**

Seventy-nine medical students were enrolled and randomly assigned to the iCST (n = 28), VR (n = 29) and iCST +VR (n = 22) groups. The study findings showed that the iCST+VR group significantly improved in cancer truth-telling perspectives at six months post-intervention. No significant difference was found between iCST and VR groups for cancer truth-telling perspectives. Although communication confidence scores significantly improved across all groups, no differences were found between groups. The iCST group reported higher learning satisfaction compared to other groups, with no significant difference between VR and iCST+VR.

**Discussion:**

This study demonstrated that VR is as effective as iCST in enhancing cancer truth-telling perspectives and communication confidence, highlighting VR’s potential as an innovative tool in medical education.

## Introduction

In the field of healthcare, effective communication has become a core competency for implementing “patient-centered care.” Medical students are not only required to receive communication skills training (CST) during their academic years but also need to continuously refine their communication abilities throughout their post-graduate year training [1]. Cancer truth-telling is one of the most frequently performed and challenging communication tasks for physicians in clinical practice. It refers to the communication of bad news in oncology. “Bad news” is defined as any information that may lead to serious consequences, such as a cancer diagnosis, recurrence and metastasis, ineffective treatment, or the absence of other curative treatment options [2]. Taiwan introduced CST for cancer truth-telling in major medical institutions nationwide, supported by funding from the Health Promotion Administration between 2010 and 2016 and promoted by the Taiwan Psycho-Oncology Society. However, a recent large-scale cross-institutional study revealed notable gaps between the actual practices of attending physicians and the truth-telling preferences of cancer patients and their families [3]. This discrepancy underscores the ongoing need to strengthen CST for healthcare providers.

Currently, there are various teaching methods for CST, including lectures, group discussions, and role-playing with simulated patients [4]. Among these methods, role-playing with simulated patients has demonstrated better learning outcomes [5,6]. The role-playing not only improves the learners’ communication abilities but also enhances their confidence in communicating with their patients [7,8]. A key concern is that the adoption of simulated patients for in-person CST (iCST) often requires substantial financial and human resources, making it both expensive and challenging to implement. It has also become impractical during the emergence of infectious disease outbreaks, such as the COVID-19 pandemic [9].

The past decade has witnessed the maturation of virtual reality (VR) in medical and nursing education [10]. VR development not only allows students to engage in self-directed learning without temporal or spatial constraints but also optimizes teaching resources. It provides a high level of realism and immersion, effectively reducing students’ apprehension and anxiety when facing unfamiliar and challenging clinical scenarios, while simultaneously enhancing their self-efficacy in performing clinical tasks. VR enables the seamless presentation, replication, and dissemination of clinical contexts that are otherwise difficult to replicate within clinical settings. Pottle [11] argued that VR offered the characteristics of repeatable standardized training, providing the medical field with an economical, secure, efficient, and scalable method that can accommodate a larger number of students, thus alleviating the instructional burden on educators. Although VR development costs can be high and time-consuming, ongoing advancements in VR technology are gradually reducing these expenses [12]. Additionally, although the use of digital technologies such as VR was accelerated during the pandemic, these virtual teaching methods have continued post-pandemic, thus signaling a shift toward a new era in medical education [13].

Current healthcare research on the application of VR focuses primarily on virtual surgery, medical diagnosis, nursing techniques, and health assessment [14,15,16]. Few studies have explored the potential of VR in facilitating medical communication training, especially on the topic of cancer truth-telling [17,18,19,20]. To bridge this gap, this study aimed to test a VR-based cancer truth-telling module to provide fifth year medical students with immersive experiences in rare clinical scenarios of cancer truth-telling within a safe and controlled environment.

## Materials and Methods

### Study Design

This experimental study assessed the effectiveness of a VR-based cancer truth-telling module. The primary outcome was students’ cancer truth-telling perspectives, and the secondary outcome was their communication confidence and learning satisfaction. Cancer truth-telling perspectives refer to students’ self-perceived awareness during the delivery of bad news regarding cancer diagnoses [21,22]. Data were collected after the participants provided their written informed consent. All participants were informed of the study’s purpose, procedures, and any potential discomfort they might experience (e.g., motion sickness) before giving their consent. Data were collected at four time-points: baseline (T0), immediately after (T1), three months after (T2) and six months after (T4) the intervention to assess the short-, mid-, and long-term benefits of the intervention, respectively. Ethical approval was obtained from the institutional review board of the study site (No. 202002415B0). Participants volunteered to participate in the study. Whether they participated or not, their right to education were not being affected. As a token of appreciation, participants who completed the survey at each time point received a local convenience store gift card valued at $100 NTD (approximately 3.6 USD).

The experiential learning model was used to develop the interventions. Experiential learning is the process of acquiring knowledge through a combination of gaining and transforming experiences [23,24]. The experiential learning model, which is a four-stage model (experiencing, reflecting, thinking, and acting), perceives learning as a cyclic process. The four stages are interdependent and work together to facilitate effective learning [24]. Various medical education programs employ this model in their interventions (e.g., role-playing) [25,26].

### Participants and Setting

This study recruited medical students from the largest medical center in Taiwan. Students who 1) were fifth-year medical students and 2) did not have motion sickness or were not prone to dizziness were eligible for this study. Participants were randomly assigned to one of the three groups: small-class in-person CST module group (iCST group), VR-based module group (VR group), and a combination of iCST and VR group (iCST+VR group). The cancer truth-telling learning process included two parts: a 30-minute video-based mini-lecture and a practical implementation. To ensure the consistency of the lecture content received by the students, this study presented a video-based mini-lecture to each group instead of a traditional face-to-face lecture.

### Theoretical Framework

This study used the SHARE model, a well-known international truth-telling theoretical framework, to develop the iCST and VR modules [27]. The framework includes four key components: setting, method of disclosure, additional information, and emotional support, and is a popular model in several CST studies in Japan and Taiwan [8,21,27]. The mini-lecture covered important aspects of the SHARE model, the preferences of Taiwanese patients with cancer and their families regarding truth-telling, common challenges, and demonstrations of the appropriate steps of the SHARE model. For the practical implementation, the iCST group engaged in the small-class in-person truth-telling CST module, the VR group received the VR-based truth-telling module, and the iCST+VR group received both the small-class iCST and VR modules.

### Interventions

#### VR-based Truth-Telling Module

To develop the VR truth-telling module, this study involved the consultation and the drawing out of expert opinions from oncologists, psychiatrists, and psychologists, and the development of three VR instructional modules reflecting the following circumstances: newly diagnosed breast cancer, recurrence and metastasis of colorectal cancer, and end-of-life lung cancer. The modules were developed in cooperation with the HTC Medical VR Department (Appendix 1). Each module incorporated both positive and negative scenarios to demonstrate appropriate and less-appropriate truth-telling performances. An “appropriate” demonstration adheres to all four aspects of the SHARE model, following best practices for truth-telling. In contrast, a “less-appropriate” demonstration omits one or more of these elements or uses inappropriate methods to disclose bad news. This intervention integrated four questions (e.g., Which of the following truth-telling performances is appropriate?) within each module to evaluate students’ abilities to identify appropriate truth-telling performances. Participants in the VR and iCST+VR groups were required to complete all three VR modules, which took them one hour to complete.

#### Small-Class In-Person Truth-Telling CST Module

The iCST module adopted from the Japan Psycho-Oncology Society (JPOS), was originally developed and implemented in a small-class format with a facilitator-to-student ratio of 2:4 [8]. It has been described in prior literature as well [21,28]. The present study employed the original iCST model in a small-class teaching setting (facilitator: students = 2:4) and implemented it in 13 groups, training 50 medical students (iCST and iCST+VR groups). Each participant in the iCST module underwent role-playing twice with simulated patients and received facilitators’ feedback concurrently. A group of the iCST module lasted three hours.

### Measurements

For a full overview of the following questionnaires in this study, see Appendix 2–4.

#### Truth-telling Questionnaire – 20 Items (TTQ-20)

The original TTQ-70, a self-report questionnaire, consists of items that have demonstrated strong reliability in previous CST studies, with Cronbach’s alpha values ranging from 0.75 to 0.91 [3,21,22]. However, due to the length and complexity of the TTQ-70, which can be burdensome in fast-paced clinical settings, this study extracted the 20-item version from TTQ-70. This 20-item version was based on a previous Taiwanese study that identified the 10 most important and 10 least important items as rated by cancer patients [3]. This study then used TTQ-20 to measure the students’ cancer truth-telling perspectives, with each item prompting them to self-rate how well they performed or perceived, including behaviors and utterances, during the intervention, on a five-point scale [27]. Higher scores indicated better cancer truth-telling perspectives [3,22]. Cronbach’s alpha of the TTQ-20 was 0.748.

#### Confidence in Communication with Patients

This 21-item confidence scale was adopted to evaluate healthcare providers’ truth-telling confidence, with each item response rated on a 10-point scale ranging from 1 (“not confident at all”) to 10 (“strongly confident”). Higher scores indicated greater confidence in truth-telling [2]. This study used the confidence scale to measure the students’ communication confidence. Cronbach’s alpha of the confidence scale was 0.970.

#### Student Satisfaction and Self-confidence in Learning Scale (SCLS)

A 13-item SCLS was used to assess students’ satisfaction and confidence in the learning activities [29], with each item response rated on a five-point scale. A higher score indicated greater satisfaction and confidence in learning activities [29]. The SCLS was used to evaluate the outcomes of various medical-simulated educational activities [30,31]. Cronbach’s alpha of the scale was 0.867.

### Statistics

The analysis employed IBM SPSS Statistics v24.0 (SPSS, Armonk, NY, IBM Corp), *p* < .05 indicating statistical significance. This study used generalized estimating equations (GEE) to analyze students’ cancer truth-telling perspectives and confidence in communication between different time-points and groups. Additionally, analysis of covariance (ANCOVA) helped compare students’ learning satisfaction between the groups. Intention-to-treat analysis was also conducted in this study.

## Results

A total of 79 participants were enrolled and randomly assigned to one of three groups: iCST (n = 28), VR (n = 29), and iCST+VR (n = 22) (Appendix 5). The average age was 23.83 ± 1.51 years. The majority of participants were male (59.5%), had a moderate-to-sufficient economic status (66.7%), and reported experiencing a moderate level of stress during their internship (66.7%). Additionally, 59.5% had taken courses related to truth-telling, and nearly all students (92.8%) expressed support for incorporating CST on truth-telling into medical education (Table 1). No participants experienced any discomfort or motion sickness while using the VR device.

**Table 1:** Demographic characteristics (N = 79).


VARIABLES		iCST (n = 28)	VR (n = 29)	iCST+VR (n = 22)	*P*

		n (%)	n (%)	n (%)	

Age, Mean (SD)		23.71 (1.72)	22.93 (1.00)	23.45 (1.71)	0.138

Sex	Male	14(50.0)	20(69.0)	14(63.6)	0.324

	Female	14(50.0)	9(31.0)	8(36.4)	

Economic status	Poor	2(7.1)	2(6.9)	3(13.5)	0.102

	Moderate	18(64.3)	14(48.3)	17(77.3)	

	Sufficient	7(25.0)	13(44.8)	1(4.5)	

Level of internship stress	Low	2(7.1)	9(31.0)	1(4.5)	**0.006**

	Moderate	14(50.0)	17(58.6)	18(81.8)	

	High	12(42.8)	3(10.3)	3(13.6)	

Has been taken courses related to truth-telling					

	Yes	17(60.7)	15(51.7)	17(77.3)	0.174

	No	11(39.3)	14(48.3)	5(22.7)	

Support to hold CST courses related to truth-telling					

	Fair	1(3.6)	1(3.4)	2(9.1)	0.901

	Support	15(53.6)	16(55.2)	11(50.0)	

	Very support	12(42.9)	12(41.4)	9(40.9)	


iCST, in-person communication skills training; CST, communication skills training; VR, virtual reality.

### Group Differences in Cancer Truth-Telling Perspectives

**Figure 1:**
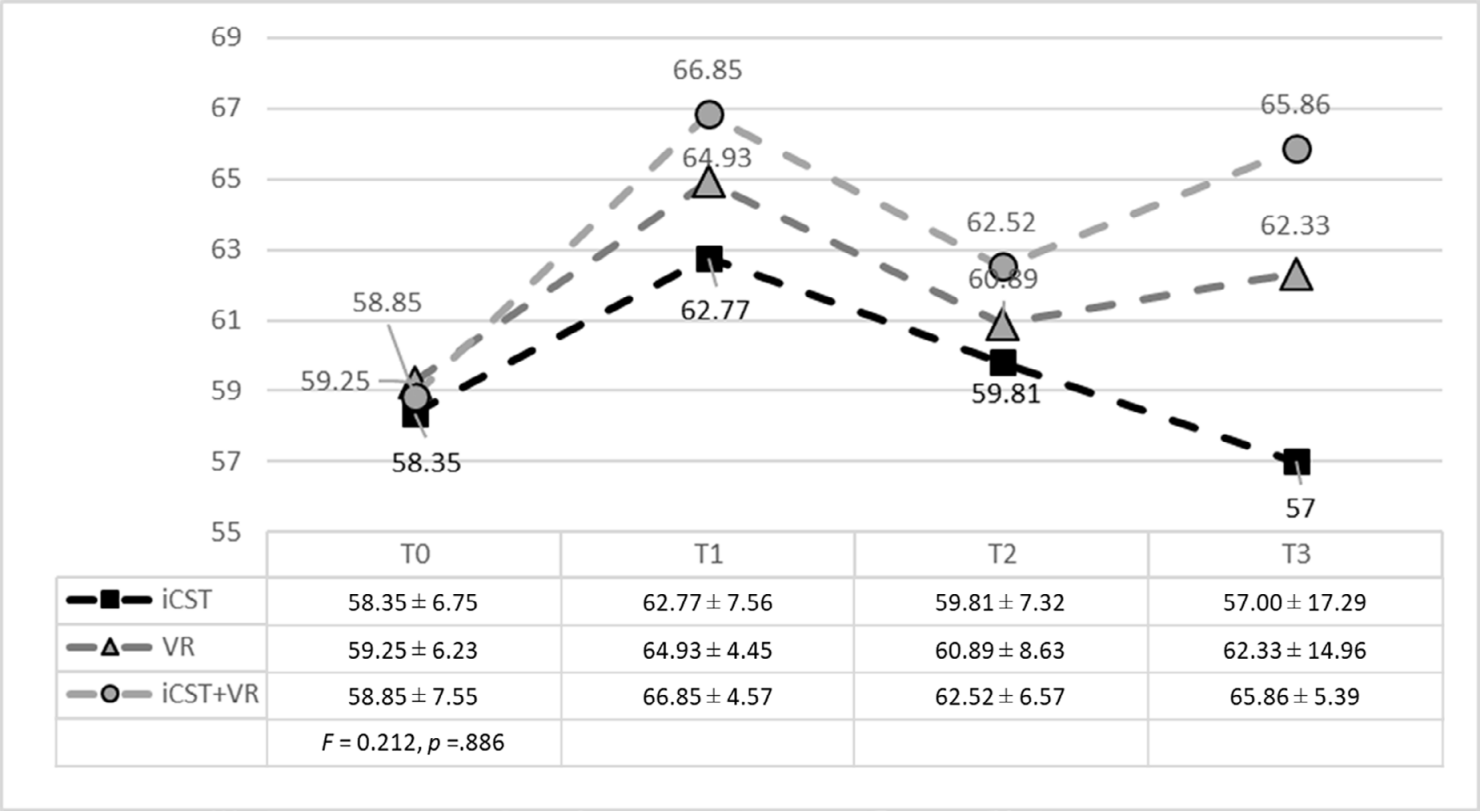
Score Trend of TTQ-20 from T0 to T3 (N = 79). TTQ-20, truth-telling questionnaire-20 items; iCST, in-person communication skills training; VR, virtual reality. T0, before the intervention; T1, immediately after the intervention; T2, three months after the intervention; T3, six months after the intervention.

There were no significant differences in TTQ-20 scores between the three groups at baseline (*F* = 0.212, *p* = .886) (Figure 1). Similarly, no significant group or time differences were found in cancer truth-telling perspectives (all *p* > .05). However, when accounting for the group × time interaction, the long-term (T3) benefit in the iCST+VR group was significantly greater than that of the other groups (*B* = 8.517, *p* = .009), with no significant differences observed in the short-term (T1) or mid-term (T2) effects (Table 2 and Figure 1).

**Table 2:** Group and Time Differences in TTQ-20 (N = 79).


VARIABLES	B	SE	WALD χ^2^	95% CI		*P*

				LOWER LIMIT	UPPER LIMIT	

Time						

T3	–1.250	2.769	0.204	–6.677	4.177	.652

T2	1.289	1.802	0.512	–2.242	4.820	.474

T1	4.036	2.169	3.461	–0.216	8.287	.063

T0	0^†^					

Group						

iCST+VR	0.341	1.971	0.030	–3.522	4.204	.863

VR	1.784	1.730	1.064	–1.607	5.176	.302

iCST	0^†^					

Group × Time						

iCST+VR × T3	8.517	3.278	6.752	2.093	14.942	**.009**

iCST+VR × T2	2.651	2.8122	0.888	–2.861	8.162	.346

iCST+VR × T1	4.313	2.8676	2.262	–1.307	9.934	.133

VR × T3	3.553	4.2521	0.698	–4.781	11.887	.403

VR × T2	–0.404	2.4621	0.027	–5.229	4.422	.870

VR × T1	0.861	2.4118	0.127	–3.866	5.588	.721

iCST × T0	0^†^					


TTQ-20, truth-telling questionnaire-20 items; iCST, in-person communication skills training; VR, virtual reality. T0, before the intervention; T1, immediately after the intervention; T2, three months after the intervention; T3, six months after the intervention. Adjusted: level of internship stress. ^†^ Reference group: iCST and T0 (baseline).

### Group Differences in Confidence in Communication

There was no significant difference in confidence scores between the three groups at baseline (*F* = 0.783, *p* = .461) (Appendix 6). Although there was a significant time difference in the confidence scores (*B* = 35.750–35.858, *p* = .000), accounting for the group × time effect yielded no significant differences in the confidence scores (*B* = 1.320–7.293, *p* = .349–.878) (Appendix 6 and Appendix 7).

### Group Differences in Learning Satisfaction

The iCST group had significantly higher learning satisfaction than the other two groups (*F* = 13.238, *p* = .000) (Appendix 8). Moreover, the post-hoc test revealed no significant difference between the VR and iCST+VR groups (mean difference = 1.548, *p* = 1.000) (Appendix 9).

## Discussion

This study found that the combination of iCST and VR modules had a significant long-term benefit for medical students, which can significantly improve students’ cancer truth-telling perspectives. The iCST+VR group likely benefitted more because the students undertook two similar truth-telling trainings (small-class iCST and VR module). Except for different practical implementations, students received the same lecture content twice, focusing on the introduction and demonstration of the SHARE model. This may provide students with a deeper understanding of the communication model, so they can perform better in cancer truth-telling perspectives.

Although prior research has confirmed significant moderate-to-high benefits of small-class iCST in improving healthcare providers’ truth-telling perceptions and confidence in communication [8,21,28], this study found no significant differences between the iCST and VR groups. This finding underscored that the effectiveness of the VR module is as good as that of the small-class iCST module. Consequently, VR can be considered as a valuable complementary tool for teaching truth-telling communication skills in medical education. Some participating students even suggested that the VR module could be used as a consolidation course after a small-class iCST. Additionally, there is a greater decrease of the cancer truth-telling perspectives score trend across the three groups, three months after the intervention. This finding suggests the importance of providing ongoing support to medical students to maintain their learning effects.

Prior studies have also confirmed that iCST can significantly improve healthcare providers’ and medical students’ communication confidence [32,33]. This study yielded findings consistent with prior studies. However, the lack of significant differences between the three groups indicates that both the small-class iCST and VR modules effectively increased the students’ communication confidence.

Regarding learning satisfaction, students who underwent the small-class iCST had higher satisfaction. The students also mentioned that some simulated patients expressed strong emotions in the role-playing. Although this scenario shocked them negatively, it provided them with a rich learning experience and helped them to learn how to deal with emotional patients. In contrast, the iCST+VR or VR groups had lower learning satisfaction, which may be due to the hardware of the VR head-mounted displays, which is still uncomfortable for students to wear for an hour. A review article indicated that during VR experiences, some users may suffer from VR sickness that is similar to motion sickness. Users may experience VR sickness even in a 10-minute virtual exposure, and the risk increases with longer exposure times [34,35]. Moreover, the Wi-Fi connection was not always stable while wearing the VR device, which may have further affected the students’ learning experiences. Fortunately, such hardware problems are expected to be resolved as artificial technology progresses.

Note that past medical educational studies applying VR technology mostly targeted hard skills (e.g., practice for operations and cardiopulmonary resuscitation) [14,15]. However, few studies have applied VR to training communication skills (soft skills) [17,18,19,20]. This study is the first to prove the effectiveness of VR in increasing medical students’ communication skills in cancer truth-telling. The cancer truth-telling perspectives of the VR group showed significant short-term improvement that decreased slightly three months after the intervention, but remained consistent six months after the intervention (Figure 1). This demonstrates the potential for sustained enhancements over time. The VR group consistently showed a significant increase in confidence scores across all time-points, underscoring the value of VR in boosting students’ communication confidence that is crucial for effective clinical practice.

### Limitations

Although this study offered valuable insights, it includes certain limitations. First, this study used a self-report measure to assess the participants’ cancer truth-telling perspectives, which may cause subjective bias. Future studies are still suggested for designing objective measures, such as using the Roter interaction analysis system to analyze video records of the intervention, to assess accurate and objective benefits. Second, a relatively small sample size and a single site design was another limitation in this study, which may have decreased the generalizability of the results. Future research with larger and more diverse participant groups will contribute to the development and testing of better communication training regarding cancer truth-telling.

### Clinical Implications

This study has significant implications for medical education. The effectiveness of the VR module suggests that immersive technologies play a valuable role in enhancing students’ cancer truth-telling perspectives and communication confidence. The findings also highlight the importance of continuous support and reinforcement for sustaining these improvements.

Although iCST has been proven effective and is systematically integrated into routine medical training, it requires substantial financial and human resources, making large-scale adoption impractical. The results emphasize the potential advantages of combining traditional iCST with VR modules. The VR module could potentially be adopted as a supplement to the iCST, as it demonstrated similar effectiveness. It would be more feasible for medical institutions to develop a module that includes a VR component and medium-sized iCST module. This improved module would benefit human resources by reducing the ratio of facilitators to students from 2:4 to 2:30. Medical educators should consider incorporating such a blended approach into their curricula to better prepare future healthcare professionals.

Meanwhile, VR is suitable for self-paced learning, and VR immersive or 3D imagery can be adopted for personalized learning. With the emerging adoption of artificial intelligence, further enhancements can be made to the combined modules. artificial intelligence technology can help enhance the learning process; it can gather objective data to assess a module’s effectiveness, and develop innovative solutions that maximize learning outcomes while minimizing costs. Furthermore, the VR-based CST modules can be adapted for other essential and challenging communication scenarios, such as shared decision-making or hospice and palliative care communication.

## Conclusions

Although iCST and VR groups had no significant difference in cancer truth-telling perspectives, this study proved that VR was as beneficial as iCST in improving students’ cancer truth-telling perspectives and communication confidence. This study contributes to medical education by highlighting the potential of innovative instructional methods, such as VR, to enhance students’ cancer truth-telling perspectives and communication confidence. Because cancer truth-telling is a common challenge for medical students, it is also recommended that the VR module be used as a consolidation course for students to repeat practice.

## Data Accessibility Statement

The data that support the findings of this study are available from the corresponding author (WRT), upon reasonable request.

## Additional File

The additional file for this article can be found as follows:

10.5334/pme.1684.s1 Appendices. Appendices 1 to 9.
