# Supplementary material for: Enhancing Cancer Truth-Telling Perspectives Using Virtual Reality in Communication Skills Training: An Experimental Study Among Medical Students
Source: Perspect Med Educ. 2025 Sep 10;14(1):539–48. doi: 10.5334/pme.1684 (PMC12428323; doi:10.5334/pme.1684)
Supplement: Appendices. — Appendices 1 to 9. [file pme-14-1-1684-s1.pdf]

Appendix 1. Development of VR-based truth-telling module

Contents	Description
**Software—Content of VR-based truth-telling module**	
Instructional modules	• *Scenarios*:<br>1. Newly diagnosed breast cancer<br>2. Recurrence and metastasis of colorectal cancer<br>3. End-of-life lung cancer<br>• *Procedure*:<br>Each module incorporated both positive and negative clinical scenarios to demonstrate appropriate and less-appropriate truth-telling performances. These scenarios demonstrated truth-telling performances by physicians.
**Hardware— Devices and Interaction process**	
Devices	• The VR modules were developed using the HTC Virti VR platform (https://app.virti.com/login).<br>• A GoPro Hero 8 camera in 360-degree mode was used to capture immersive video footage for the 3D environment.<br>• Students accessed the truth-telling modules using the HTC VIVE Focus 3 headset.
Assess	The VR module was a web-based and hosted on the HTC Virti platform, requiring students to connect to Wi-Fi for access.
Interaction	• Students interacted with the VR modules via a headset.<br>• Within each module, students were prompted to answer four pre-set questions designed by the research team to assess their understanding and reflection.
Collaoration team	HTC Medical VR Department<br>(This study collaborated with a teaching manager from HTC medical education to assist in developing and filming the cancer truth-telling VR module, ensuring their quality and feasibility.)

VR, vitural reality.

Appendix 2. Truth-telling questionnaire – 20 Items (TTQ-20)

To better understand your actual perspectives in cancer truth-telling, as a reference for future medical education, we kindly ask you to answer the following questions. There are no right or wrong answers—Please check the option that best best reflects your thoughts. Your answer is of great significance to the training of cancer truth-telling. Thank you for your assistance!

The response options are as follows:

1 = Strongly Disagree	2 = Disagree	3 = Neutral
4 = Agree	5 = Strongly Agree	

Items	Actual perspectives				
1. Looking at the patient's eyes and face	1	2	3	4	5
2. Telling about the patient's life expectancy	1	2	3	4	5
3. Telling the treatment plan	1	2	3	4	5
4. Telling the recommended treatment	1	2	3	4	5
5. Breaking bad news only to the patient	1	2	3	4	5
6. Breaking bad news by telephone	1	2	3	4	5
7. Breaking bad news in a vague manner	1	2	3	4	5
8. Telling only bad news	1	2	3	4	5
9. Answering the patient's questions	1	2	3	4	5

Items	Actual perspectives				
10. Communicating clearly the main points of bad news	1	2	3	4	5
11. Breaking bad news in a setting with family	1	2	3	4	5
12. Talking gently	1	2	3	4	5
13. Writing on paper to explain	1	2	3	4	5
14. Giving papers that physician referred to	1	2	3	4	5
15. Physician deciding on the method of treatment	1	2	3	4	5
16. Breaking bad news honestly	1	2	3	4	5
17. Breaking bad news in a way that is easy to understand	1	2	3	4	5
18. Dealing with the patient's questions in an irritated manner	1	2	3	4	5
19. Breaking bad news before it is definite	1	2	3	4	5
20. Talking in a business-like manner	1	2	3	4	5

Appendix 3. Confidence in communication with patients

In order to understand your self-confidence in cancer truth-telling, please answer the following questions. Please fill in the answer based on "your current self-confidence." A score of 1-point indicates no confidence at all, and 10-point indicates very confident. The answer doesn't matter whether it is right or wrong. Please circle out the most suitable option. Your answer is of great significance to the training of cancer truth-telling. Thank you for your assistance!

Items	No confidence at all → Very confident									
1. Creating comfortable setting	1	2	3	4	5	6	7	8	9	10
2. Assessing patient's ability to discuss bad news	1	2	3	4	5	6	7	8	9	10
3. Detecting verbal cues	1	2	3	4	5	6	7	8	9	10
4. Encouraging family presence	1	2	3	4	5	6	7	8	9	10
5. Assessing current knowledge	1	2	3	4	5	6	7	8	9	10
6. Detecting patient's anger	1	2	3	4	5	6	7	8	9	10
7. Including family in discussion	1	2	3	4	5	6	7	8	9	10
8. Detecting nonverbal cues	1	2	3	4	5	6	7	8	9	10
9. Assessing how much the patient wants to know	1	2	3	4	5	6	7	8	9	10
10. Detecting anxiety	1	2	3	4	5	6	7	8	9	10

Items	No confidence at all				→					Very confident
11. Planning discussion in advance	1	2	3	4	5	6	7	8	9	10
12. Detecting patient's sadness	1	2	3	4	5	6	7	8	9	10
13. Confirming patient's understanding of cancer	1	2	3	4	5	6	7	8	9	10
14. Checking to see that information was received accurately by patient	1	2	3	4	5	6	7	8	9	10
15. Providing information in small increments	1	2	3	4	5	6	7	8	9	10
16. Avoiding medical jargon	1	2	3	4	5	6	7	8	9	10
17. Reinforcing and clarifying information	1	2	3	4	5	6	7	8	9	10
18. Responding empathetically to patient's feelings	1	2	3	4	5	6	7	8	9	10
19. Planning a strategy for disclosing information	1	2	3	4	5	6	7	8	9	10
20. Handling patient's emotional reactions	1	2	3	4	5	6	7	8	9	10
21. Managing your own response to patient distress	1	2	3	4	5	6	7	8	9	10

Appendix 4. Student satisfaction and self-confidence in learning scale (SCLS)

To better understand your learning satisfaction and confidence in this training, as a reference for future cancer truth-telling education, we kindly ask you to answer the following questions. There are no right or wrong answers—Please select the option that best reflects your thoughts. Your answer is of great significance to the training of cancer truth-telling. Thank you for your assistance!

The response options are as follows:

1 = Strongly Disagree	2 = Disagree	3 = Neutral
4 = Agree	5 = Strongly Agree	

Items	Your responses				
1. The teaching methods used in this training were helpful and effective.	1	2	3	4	5
2. The training provided me with a variety of learning materials and activities to promote my learning the cancer truth-telling communication skills.	1	2	3	4	5
3. I enjoyed how my instructor taught the training.	1	2	3	4	5
4. The teaching materials used in this training were motivating and helped me to learn.	1	2	3	4	5
5. The way my instructor(s) taught the training was suitable to the way I learn.	1	2	3	4	5
6. I am confident that I am mastering the content of the training activity that my instructors presented to me.	1	2	3	4	5
7. I am confident that this training covered critical content necessary for the mastery of cancer truth-telling.	1	2	3	4	5

Items	Your responses				
8. I am confident that I am developing the skills and obtaining the required knowledge from this training to perform necessary tasks in a clinical setting.	1	2	3	4	5
9. My instructors used helpful resources to teach the training.	1	2	3	4	5
10. It is my responsibility as the student to learn what I need to know from this training activity.	1	2	3	4	5
11. I know how to get help when I do not understand the concepts covered in the training.	1	2	3	4	5
12. I know how to use training activities to learn critical aspects of these skills.	1	2	3	4	5
13. It is the instructor's responsibility to tell me what I need to learn of the training activity content during class time.	1	2	3	4	5

Appendix 5. **CONSORT Flow Chart**

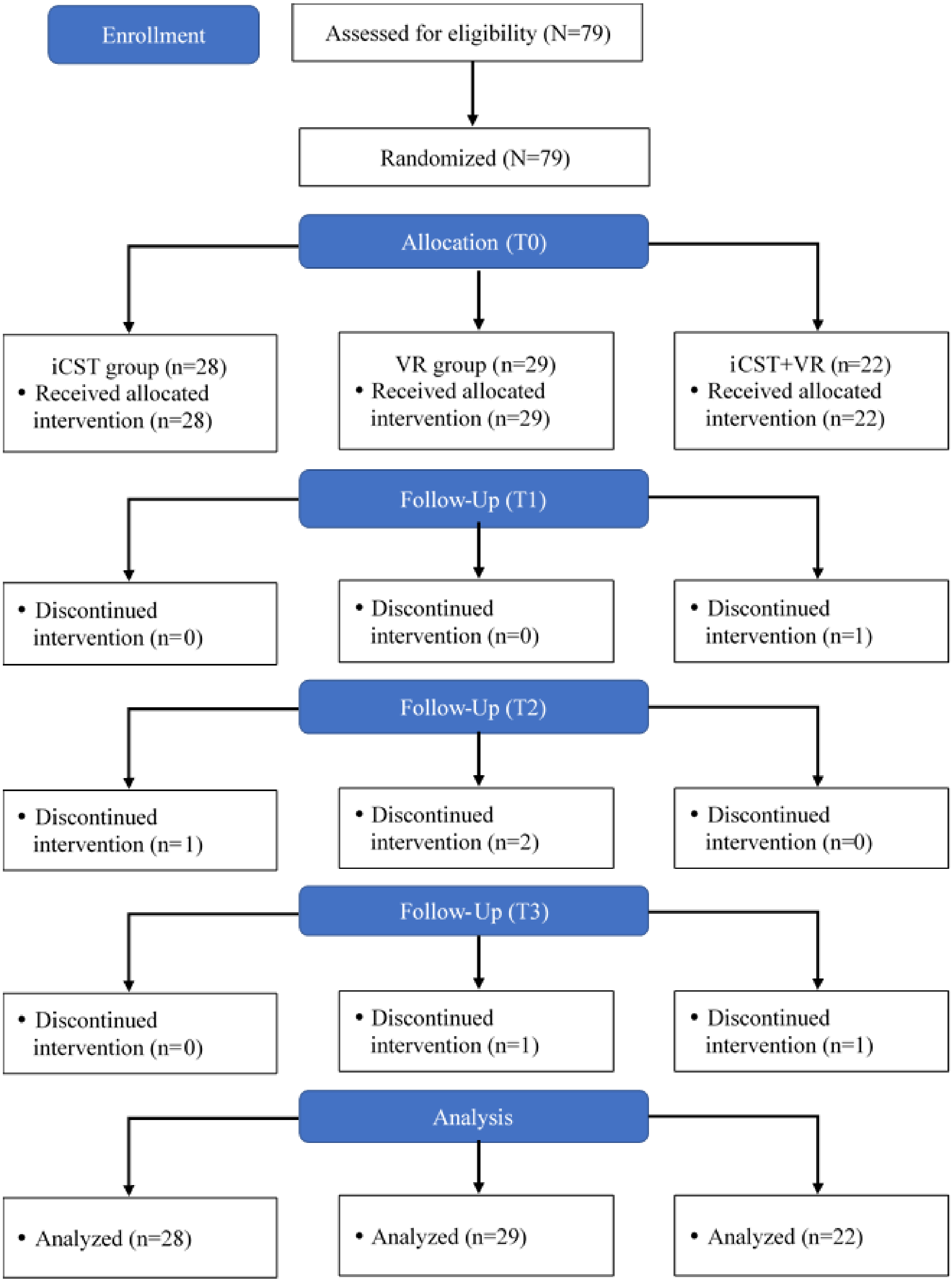

iCST, in-person communication skills training; VR, virtual reality.

Appendix 6. Score Trends of Confidence in Communication from T0 to T3 (N=79)

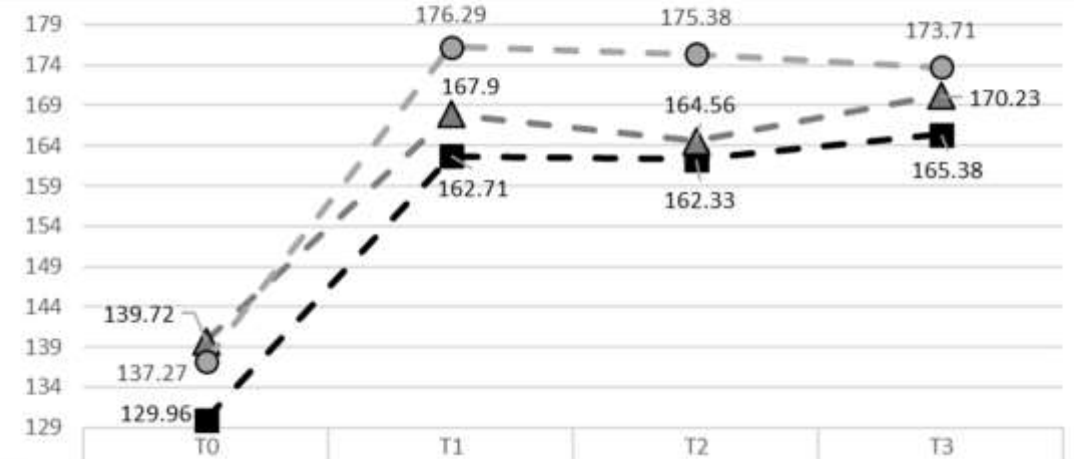

	T0	T1	T2	T3
iCST	129.96±26.85	162.71±29.78	162.33±17.72	165.38±22.20
VR	139.72±27.38	167.90±21.74	164.56±23.11	170.23±24.60
iCST+VR	137.27±37.57	176.29±20.35	175.38±19.34	173.71±25.35
	$F = 0.783, p = .461$			

iCST, in-person communication skills training; VR, virtual reality.

T0, before the intervention; T1, immediately after the intervention; T2, three months after the intervention; T3, six months after the intervention.

Appendix 7. Group and Time Differences in Communication in Confidence (N=79)

Variables	B	SE	Wald χ^2	95% CI Lower limit	95% CI Upper limit	*p*
Time						
T3	35.780	5.0054	51.098	25.970	45.591	.000
T2	32.858	4.2228	60.546	24.582	41.134	.000
T1	32.750	4.8801	45.037	23.185	42.315	.000
T0	0†					
Group						
iCST+VR	7.308	9.2796	0.620	-10.879	25.496	.431
VR	9.760	7.0570	1.913	-4.072	23.591	.167
iCST	0†					
Group x Time						
iCST+VR x T3	1.320	8.6345	0.023	-15.603	18.244	.878
iCST+VR x T2	6.263	8.0039	0.612	-9.424	21.951	.434
iCST+VR x T1	7.293	7.7938	0.876	-7.982	22.569	.349
VR x T3	-5.121	6.4761	0.625	-17.814	7.572	.429
VR x T2	-7.925	6.1552	1.658	-19.989	4.139	.198
VR x T1	-4.578	5.7167	0.641	-15.782	6.627	.423
iCST x T0	0†					

iCST, in-person communication skills training; VR, virtual reality. T0, before the intervention; T1, immediately after the intervention; T2, three months after the intervention; T3, six months after the intervention.

Adjusted: level of internship stress. † Reference group: **iCST** and T0 (baseline).

Appendix 8. Score Distribution of Secondary outcome—Student Satisfaction and Confidence in the Learning Activities (N=79)

**Variables**	**Time**	**iCST (n=28)**	**VR (n=29)**	**iCST+VR (n=22)**	***F***	***p***
		**Mean (SD)**	**Mean (SD)**	**Mean (SD)**		
SCLS †	T1	62.07 (3.41)	54.03 (6.08)	52.29 (11.10)	13.238	.000

iCST, in-person communication skills training; VR, virtual reality; SCLS, student satisfaction and self-confidence in learning scale.

T1, immediately after the intervention.

† Analyze using ANCOVA and adjusted level of internship stress.

Appendix 9. Post hoc test for SCLS between iCST, VR, and iCST+VR group (N=79)

Group	Mean difference	95% CI		*p*
		Lower limit	Upper limit	
iCST vs. VR	8.402	3.351	13.453	.000
iCST vs. iCST+VR	9.950	4.804	15.096	.000
VR vs. iCST+VR	1.548	-3.599	6.694	1.000

SCLS, student satisfaction and self-confidence in learning scale; iCST, in-person communication skills training; VR, virtual reality.
